# Differential Release of Exocytosis Marker Dyes Indicates Stimulation-Dependent Regulation of Synaptic Activity

**DOI:** 10.3389/fnins.2019.01047

**Published:** 2019-10-02

**Authors:** Andreas W. Henkel, Abdeslam Mouihate, Oliver Welzel

**Affiliations:** ^1^Department of Physiology, Faculty of Medicine, Kuwait University, Kuwait City, Kuwait; ^2^Department of Psychiatry and Psychotherapy, University Hospital Erlangen, Erlangen, Germany

**Keywords:** hippocampal neurons, exocytosis, fusion pore, FM1-43, kiss-and-run

## Abstract

There is a general consensus that synaptic vesicular release by a full collapse process is the primary machinery of synaptic transmission. However, competing view suggests that synaptic vesicular release operates via a kiss-and-run mechanism. By monitoring the release dynamics of a synaptic vesicular marker, FM1-43 from individual synapses in hippocampal neurons, we found evidence that the release of synaptic vesicle was delayed by several seconds after the start of field stimulation. This phenomenon was associated with modified opening kinetics of fusion pores. Detailed analysis revealed that some synapses were completely inactive for a few seconds after stimulation, despite immediate calcium influx. This delay in vesicular release was modulated by various stimulation protocols and different frequencies, indicating an activity-dependent regulation mechanism for neurotransmitter exocytosis. Staurosporine, a drug known to induce “kiss-and-run” exocytosis, increased the proportion of delayed synapses as well as the delay duration, while fluoxetine acted contrarily. Besides being a serotonin reuptake inhibitor, it directly enhanced vesicle mobilization and reduced synaptic fatigue. Exocytosis was never delayed, when it was monitored with pH-sensitive probes, synaptopHlourin and αSyt-CypHerE5 antibody, indicating an instantaneous formation of a fusion pore that allowed rapid equilibration of vesicular lumenal pH but prevented FM1-43 release because of its slow dissociation from the inner vesicular membrane. Our observations suggest that synapses operate via a sequential “kiss-and-run” and “full-collapse” exocytosis mechanism. The initially narrow vesicular pore allows the equilibration of intravesicular pH which then progresses toward full fusion, causing FM1-43 release.

## Introduction

The predominant neurotransmitter exocytosis mechanism “*kiss-and-run*” or “*full-collapse fusion*” (Heuser and Reese, 1973; Ceccarelli and Hurlbut, 1980) in the brain is still under debate, despite a wealth of evidence collected for both models in many different cell- and vesicle types (Albillos et al., 1997; Schikorski and Stevens, 2001; Richards et al., 2005). While it is widely accepted that both mechanisms occur in mast- and neuro-endocrine cells as well as large dense core vesicles (Elhamdani et al., 2006; Xia et al., 2009; Balseiro-Gomez et al., 2016), either as separate mechanisms or in combinations of both, their functional significance for CNS-synapses with small synaptic vesicles is much less conclusive (Harata et al., 2006; Granseth et al., 2009; Zhang et al., 2009; Rudling et al., 2018).

It is not clear yet, whether the variation in the process of vesicular release is due to the difference in types of synapses (GABAergic, glutamatergic, etc.), difference in the regulation of release of two and more transmitters from a single synapse (Jonas et al., 1998), or the change in the exocytosis kinetics of a specific synapse class (Gan and Watanabe, 2018). Many research groups have shown that information transfer between neurons can be modulated by regulating the release of presynaptic neurotransmitter and or by affecting postsynaptic receptor densities, leading to such phenomena as LTP and LTD (Castillo, 2012). These features enable a dynamic processing of information, reaching far beyond classical linear information transfer and rather may form the basis of complex cognitive functions (Fernandes and Carvalho, 2016). We have observed that individual synapses in hippocampal cultures exhibit a large heterogeneity of release kinetics that may add a temporal component to the synaptic information processing mechanism (Henkel, 2015).

Individual synapses, even of the same class, are able to adjust neurotransmission not only quantitatively but also temporally. The mechanisms underlying highly dynamic variation of synaptic vesicle release have been shown to be linked to different vesicular pools, variable modes of mobilization, docking access, calcium levels, protein phosphorylation (Petrov et al., 2015), and a variety of endocytosis mechanisms.

A detailed view on the exocytosis of individual vesicles originates mostly from electrophysiological experiments on large granules, since their fusion process could directly be monitored with “cell-attached” capacitance measurements (Albillos et al., 1997; Henkel et al., 2000). Smaller vesicles (<50 nm in diameter) are much harder to analyze in detail by electrophysiological techniques due to technical limitations. One exception is the large synapse “Calyx of Held,” where single vesicle fusions have been resolved and analyzed (He et al., 2006).

Much of our knowledge was compiled from indirect exo- and endocytosis analyses with fluorescent activity-dependent dyes (Betz et al., 1992; Harata et al., 2006). These optical techniques allowed the simultaneous monitoring of many synapses (200–1500 synaptic buttons per image frame) in hippocampal cultures (Klingauf et al., 1998). The synapses do not necessarily belong to a homogenous neurotransmitter class but were rather composed of different synapse types, most of them being glutamatergic (Megias et al., 2001). We addressed the question of whether the release kinetics of FM1-43 in a selected individual synapse was constant or dynamic under changing stimulation conditions. In particular, we focused on the mechanistic kinetic process that affected the delay of dye release in individual synapses under increasingly stronger stimulation and concurrent application of fluoxetine or staurosporine (Henkel, 2015). The non-specific kinase inhibitor staurosporine was used to test which exo-endocytosis mechanism (“classical exocytosis” or “kiss-and run”) was operating at synapses under different conditions (Henkel and Betz, 1995; Klingauf et al., 1998; Henkel et al., 2000). We had previously shown that fluoxetine could overcome synaptic fatigue after excessive stimulation (Henkel et al., 2010). But since the mechanism remained unknown, we used a selection of activity-dependent staining techniques and drugs to investigate variability, timing, and kinetic mechanisms of exo- and endocytosis in individually visualized synapses in order to experimentally document a consistent model of vesicle fusion.

## Materials and Methods

### Experiments and Animals

Experiments were conducted on primary cell cultures, obtained from 2- to 4-day-old Wistar rats (supplied by the Animal Resource Center in Kuwait and the Franz-Penzoldt-Zentrum, Erlangen, Germany) of both sexes, at the laboratories of the Psychiatric University Clinic in Erlangen, Germany, the Research Core Facility at the Kuwait University and the laboratories of the Department of Physiology, Kuwait University. We decided to use neonatal neuron preparation with fully functional synapses because it was not possible to obtain healthy functional synapses from neurons of adult rats. All animal work conducted in Erlangen was approved by the Kollegiales Leitungsgremium of the Franz-Penzoldt Zentrum, Erlangen, Germany, in accordance with the Animal Protection Law of the Federal Republic of Germany and Animal care while all handling procedures conducted in Kuwait complied with standards of the International Council of Laboratory Animals Sciences and were approved by the Kuwait University Research Administration Ethics Committee.

### Cell Culture

Neonatal hippocampal neurons and associated glial cells were cultured as described (Klingauf et al., 1998; Welzel et al., 2011) with minor modifications. In brief, newborn rats were sacrificed by decapitation, whole hippocampi were removed from the brain, transferred into ice-cold Hank’s salt solution, and cleaned from adhesive tissue and blood vessels. After digestion with trypsin (5 mg/mL), cells were triturated in a glass pipette, and plated in minimal essential medium (MEM), supplemented with 10% fetal calf serum and 2% B27 Supplement (all from Invitrogen, Karlsruhe, Germany) on 18 mm glass coverslips, coated with Matrigel (BD Biosciences, San Jose, CA, United States). The coverslips were placed in 24-well plates and the plates were wrapped with cling wrap to maintain iso-osmolarity (320 mOsmol). The wrapping allowed diffusion of gases like CO_2_ but retained evaporated water. Antibiotic/antimycotic (Gibco, Grant Island, United States) solution was added. Cells were used between 20 and 30 days in culture.

### Transfection With SynaptopHlourin

To monitor synaptic vesicle exocytosis by measuring the collapse of the vesicular proton gradient, neurons were transfected with synaptopHlourin, a pH-sensitive GFP variant that was fused to the lumenal domain of synaptobrevin (VAMP), controlled with a synapsin promoter (Sankaranarayanan et al., 2000). Transfection was done on the 3rd day *in vitro* with a modified calcium phosphate technique, described elsewhere (Threadgill et al., 1997; Welzel et al., 2010).

### FM1-43 Loading, Destaining, and Imaging

Cells, attached to 18 mm cover slips, were transferred to a stimulation chamber, containing rat Ringer’s solution (in mM): NaCl 145, KCl 4, MgCl_2_ 2.5, CaCl_2_ 2.5, glucose 10, and HEPES 10, pH 7.2, 300 mOsmol, mounted on the microscope stage and experiments were conducted for up to 45 min at room temperature 22–24°C. The experimental protocol was employed for activity-dependent vesicle staining and subsequent measuring of exocytosis. Synapses were loaded by electrical stimuli trains at 30 Hz for 20–30 s with 2.5 μM FM1-43 (Thermo Fisher Scientific, Waltham, MA, United States), dissolved in the Ringer’s solution as previously described (Henkel et al., 2010). Stimulation was administered through two parallel platinum wires, spaced 1 cm apart. Supplementary Figure S1 depicts the extended experimental “sequential loading procedure,” used in some experiments. The preparation was stimulated with 1 ms bipolar current pulses (50 mA) at 30 Hz for 30 s, in the presence of FM1-43 for loading synaptic vesicles. The solution containing FM1-43 was immediately removed, briefly rinsed, and washed six times for 1 min. The preparation was illuminated for 30 s with 30 flashes for 250 ms each, to attenuate strong initial bleaching. Destaining was triggered by stimulation at 30 Hz for 20 s. Images were taken from 10 s before the start of the stimulation to the end at 1 image/s, 250 ms exposition time. In the case of sequential staining procedures, the staining and destaining sequence was repeated as exemplified in Supplementary Figure S1. Drugs [staurosporine, 2 μM (Calbiochem, United States); fluoxetine, 10 μM (Sigma-Aldrich, United States)] were either applied 30–60 min before the experiments or in between the “sequential loading procedure” for 10 min as indicated and were present in all subsequent washing and stimulation media. In control experiments, drugs were omitted and experiments were conducted as described above.

### Labeling Synapses With αSyt1-CypHerE5 Antibody

αSyt1-CypHer5E (Synaptic Systems, Göttingen, Germay) is an antibody against the vesicle-specific protein synaptotagmin, coupled to a modified, pH−sensitive derivative of Cy5 (Adie et al., 2002) that permanently labels the lumen of recycled synaptic vesicles. In contrast to synaptopHlourin, its fluorescence is quenched at pH ∼7.2 and becomes brighter once transferred inside synaptic vesicles at pH < 5.5. Synapses were loaded with αSyt1-CypHer5E (50 μg/ml) by incubation in high K^+^-Ringer’s (40 mM) for 10 min at 37°C, washed two times with normal Ringer’s, and were quickly imaged thereafter. The preparation could be stimulated several times at 30 Hz, since the fluorescence decay recovered after endocytosis and re-acidification of vesicles. Fluorescence was monitored on a TI-Eclipse inverted microscope (Nikon, Melville, United States), using a Nikon Cy5 filter set (excitation: 604–644 nm, emission: 672–712 nm).

### Calcium Measurement

The experiments were conducted on the 25th day *in vitro*. Cells, grown on 18 mm cover slips, were loaded with 5 μM Ca-GreenAM (Thermo Fisher Scientific, Waltham, MA, United States) or 1 μM Fura-2AM (Calbiochem- now Merck, Darmstadt, Germany), dissolved in Modified Eagle’s Medium (MEM) for 90 min at 37°C. Cells were then transferred to an electrical stimulation chamber, mounted on the microscope stage, and stimulated with trains of bidirectional 1 ms pulses at 30 Hz for 30 s. The Ca-GreenAM signal was imaged by a conventional FITC filter set (490 nm excitation/520 nm emission) while Fura-2 fluorescence was monitored using filter set with 380 nm excitation and 520 nm emission. No quantitative 340/380 nm ratio-metric measurement was used for Fura-2 excitation.

### Image Quantification and Data Analysis

Images were processed and analyzed using a package of self-written programs for image processing and data analysis: “Image Processing and Data Analyzing Software” (IPDAS, SynoSoft, Kuwait) (Henkel et al., 2014; Mouihate et al., 2019).

At first, fluorescent peaks corresponding to synapses and non-synaptic artifact spots were detected and their intensity course was quantified in all frames by ImageStar V. 2.18 software, SynoSoft. The peak detection algorithm excluded hazy areas and overlapping or odd-shaped spots, but included inactive (not destaining) spots. Spot detection parameters included a general intensity threshold, a shape parameter fit, a local background detection threshold, and a peak density-minimum-distance threshold, all build in ImageStar.

The individual fluorescence intensity traces from all these spots were stored and further analyzed by the self-written program “Exocytosis Analysis,” SynoExA, Ver. 1.36. SynoExA selected only fluorescence traces that met minimum data quality criteria, which included noise level (rms noise < 1.5), trace course (exclusion of brighter growing and wave-like traces), and bleach correction, derived from pre- and post-stimulation intensity fluorescence sections. A trace was defined as synaptic, when the difference between pre- and post-stimulation regression lines at the mid-stimulation period was >0.1 d*F*/*F*_*max*_ of the initial fluorescence (*F*_*max*_). These regression lines were also used to calculate the fluorescence loss during stimulation. Fluorescence change of each individual spot was quantified from the start to the end of the stimulation period and were expressed as normalized change (d*F*/*F*_*max*_) (Henkel et al., 2010). Synapses, whose d*F*/*F*_*max*_ was larger than 0.35 were defined as very active synapses (VAS).

Delay release time (*D*_*t*_) was defined as the time difference between start of stimulation (Stim_0_) and the onset of dye release on low-pass filtered synaptic traces. The threshold for release onset was arbitrarily defined as the time, when the initial fluorescence (d*F*/*F*_*max*_) dropped below 4% of its pre-stimulation value. A synapse was designated as “delayed,” if its fluorescence decrease was <4% within 5 s after stimulation onset.

The software was also used to select traces according to defined kinetic properties and assigned them to specific synaptic classes. Threshold settings for selection parameter were defined by the user and indicated in the section “Results.” Only traces derived from spots without increasing intensities, which were below a minimum noise level of 1.5 rms and showed sufficient trace steadiness (no wave-like traces), were included in the data analysis. Table 1 lists the available class selection criteria, implemented into SynoExA.

**TABLE 1:** Synaptic class definitions.

1. Active synapses, mono-exponential decay, [d*F*/*F*_*max*_], >0.1 = active synapses, [d*F*/*F*_*max*_], >0.35 = VAS.
2. Delayed synapses, onset of fluorescence release by stimulation [*D*_*t*_].
3. Fast destaining synapses.
4. Linear synapses, defined by constant linear release rate.
5. Two-speed-component synapses with double exponential decay function.

Several kinetic parameters were determined in each of the synapse classes to characterize class-specific release properties. Table 2 lists all available parameters.

**TABLE 2:** Kinetic parameter for synapse characterization.

Initial fluorescence intensity (*F*_*max*_) (range: 0–255)
Absolute fluorescence release (d*F*) (range: 0–255)
Relative fluorescence release (d*F*/*F*_*max*_) (range: 0–1)
Half-maximal release time (tau_50_) (s)
Fast release, defined by maximal release velocity (*V*_*max*_) (d*F*)
Fast release, defined by initial release slope
Time of *V*_*max*_ after stimulation start (*t*−*V*_*max*_) (s)
Fluorescence release delay after stimulation start (s)
Linearity of release (linear fit regression coefficient, linear regression correlation > 0.997)
Percentage of active synapses (%)

### Statistics

Statistical analysis was performed with SynoStat 1.23 (SynoSoft) and Microsoft Excel 2016 (Microsoft). Kolmogorov–Smirnov test and Shapiro–Wilk test were used to check if the data followed a normal distribution. One-way ANOVA analyses were performed if more than two conditions were compared.

Results from single data series were expressed as *mean* ± *standard deviation*. Results, derived from data series, collected from repeated parallel experiments were expressed as *weighted mean* (${\bar{x}}_{w}$) ± *weighted standard deviation* (*sd*_*w*_), to account for different synapse numbers in independent experiments. Weights (*w*_*i*_) were set according to their relative contribution (number of synapses) in individual experiments; *n* = number of experiments; *m* = number of non-zero weights.

$${\bar{x}}_{w}=\frac{\sum_{i=1}^{n}w_{i}\,x_{i}}{\sum_{i=1}^{n}w_{i}}$$

$$s\,d_{w}=\sqrt{\frac{\sum_{i=1}^{n}w_{i}\,{\left(x_{i}-{\bar{x}}_{i}\right)}^{2}}{\sum_{i=1}^{n}w_{i}\,\frac{\left(m-1\right)}{m}}}$$

Normally distributed data were tested for significant differences between groups with Student’s *t*-test, where the significance acceptance level was set to *p* < 0.05. To assess the effect size of an experimental intervention, Cohen’s *D* was calculated to compare experimental groups. The effects size was determined to:

0 < 0.2 “no effect,” 0.2–0.3 “small effect,” around 0.5 “medium effect,” >0.8 “large effect” (Cohen, 1988).

Categorical data for two population proportions, like differences between groups, expressed as percentages, were tested for significance with the *Z*-test, where the significance acceptance level was set to *p* < 0.05.

## Results

### Kinetically Different Synapse Classes in Hippocampal Preparations

**FIGURE 1:**
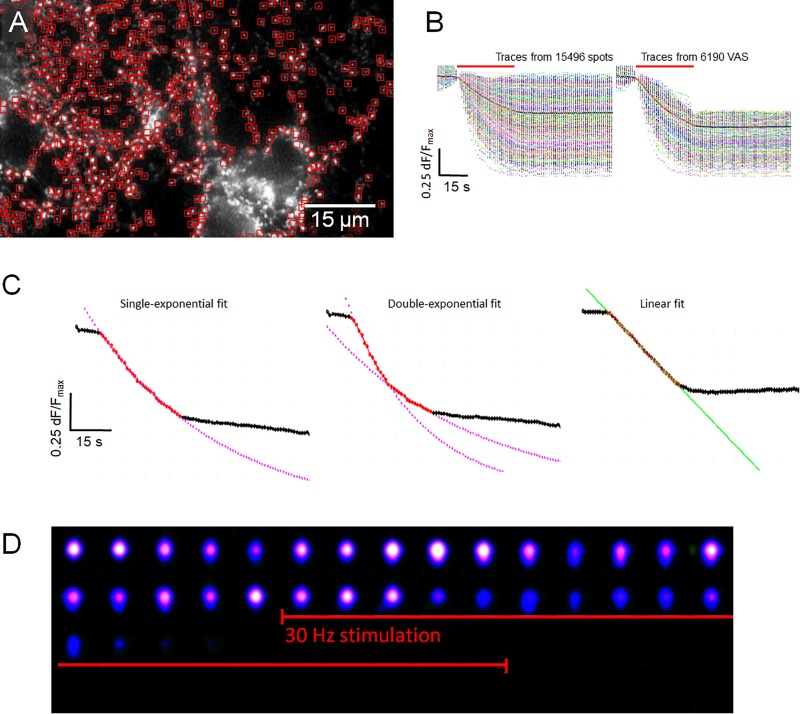
**(A)** Three examples of synaptic classes, selected by SynoExa according to specified kinetic courses and fitting functions were overlaid onto mean traces during the stimulation period. Left panel: “Classical synapses” derived from *n* = 453 individual synapses, *r* = 0.997; middle panel: “Two-speed synapses,” *n* = 342, tau_1_: *r* = 0.998, tau_2_: *r* = 0.999; and right panel: “Linear synapses,” *n* = 126, *r* = 0.999. **(B)** Pseudo-colored image series (imaging rate: 1 Hz) shows a single delayed synapse that started destaining 3 s after start of stimulation (red bar). **(C)** Hippocampal synapses, stained with FM1-43. Individual spot intensities were detected and extracted with ImageStar (red box markers), and stored in a database together with their *x*–*y* positions. **(D)** Fluorescent traces, derived from individual spots, normalized to start of stimulation, overlaid onto the mean trace (black–red–black). Left panel, Traces from all identified spots; right panel, selected traces from synapses (VAS) with >0.35 d*F*/*F*_*max*_ FM1-43 release.

The kinetics of FM1-43 release from individual synaptic boutons appeared to be highly variable although the average of several hundreds of fluorescence traces generally followed a single or second-order exponential decay function, which was the result of mutual compensatory effects of fast and slow destaining synapses. Destaining patterns of individual synapses were analyzed in high detail and three main kinetic classes were defined, namely: (1) “Classical synapses” showed a dye release kinetic that followed a single exponential decay function, (2) “Two-speed synapses,” characterized by a double exponential decay function, and (3) “Linear synapses” exhibiting a constant release rate. A special type of synapses was discovered and defined as “Delayed synapse” (DelSyn), whose release kinetics belonged to either of the classes’ (1–3), but whose onset of release was considerably delayed after the start of the stimulation. Representative examples of the three defined synaptic kinetic classes are shown in Figure 1A, while Figure 1B presented an example of a DelSyn. The depicted traces were averaged from individual synapses, extracted from the original fluorescent spot database by SynoExA. The initial challenge for this study was the reliability for detection of synapses from all fluorescently FM1-43 stained objects, a task that was conducted by “ImageStar” Ver. 2.18. Figure 1C shows a typical hippocampal preparation after an activity-dependent FM1-43 staining, marking all detected round or oval shaped spots that laid outside high background areas. The intensity traces derived from all detected spots were separately analyzed, as depicted in the left panel of Figure 1D, and traces were defined as “VAS,” if they followed a large fluorescence decrease (d*F*/*F*_*max*_ > 0.35) during the stimulation period (right panel).

### A Subpopulation of VAS Shows Delayed Release of FM1-43

**FIGURE 2:**
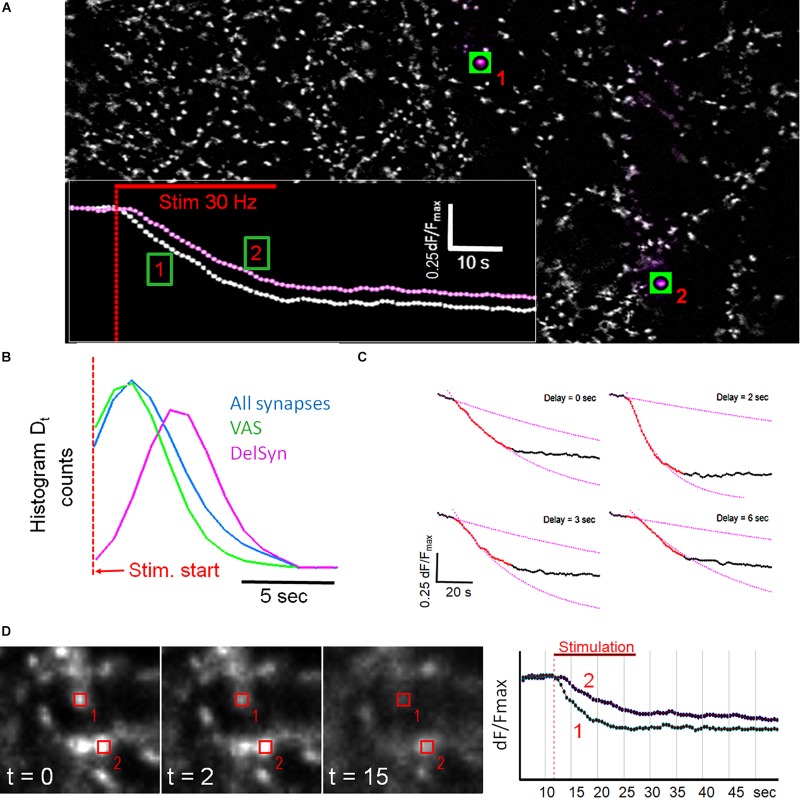
**(A)** Fluorescent spots from Figure 1C after background subtraction. The traces from two labeled synapses are shown in the inset. Inset: classical synapse (1) and a delayed onset synapse (2). **(B)** Histogram of first-order derivatives of mean traces indicate *D*_*t*_ distribution of presented kinetic subpopulations as indicated by color. **(C)** Four panels showing individual synaptic fluorescence traces with increasing *D*_*t*_ and corresponding single exponential fits to trace sections after the start of FM1-43 release. Pre-stimulation sections were fitted with linear regression lines. **(D)** Comparison of two neighboring synapses with different *D*_*t*_. The graph shows overlaid traces from regions, indicated in the images.

Figure 2A shows that fluorescent spots, after background subtraction by a deconvolution filter, exhibited a wide variability in morphological shapes and fluorescence intensities. The spot traces were also broadly different with regard to release velocity, release quantity, kinetic course, and onset of destaining. Traces of regular and DelSyns could be clearly distinguished, when their traces were directly overlaid (inset in Figure 2A). DelSyns were extracted from a dataset of VAS (*n* = 242, from 10 experiments, d*F*/*F*_*max*_ > 0.35) and analyzed in detail. It was found that the distribution of *D*_*t*_ could be fitted by a Gaussian bell curve with a weighted mean of 4.75 ± 1.52 s, ranging from 1 to 10 s). When *D*_*t*_ was measured on all synaptic traces, *D*_*t*_ distribution was shifted to about half this value, while the VAS subset showed a reduction of long *D*_*ts*_, as demonstrated in Figure 2B. Figure 2C shows typical examples of individual synaptic fluorescence traces with increasing *D*_*t*_ and how *D*_*t*_ was measured and visualized at the intersection of a linear regression line that was fitted to the pre-stimulation section and a single exponential decay function to a 10 s section after the start of fluorescence decay.

Spatial analysis revealed that fluorescent spots were not evenly distributed but formed chain-like structures and large clusters on cell aggregates (Supplementary Figure S2). Although DelSyns were mostly concentrated in clusters, they were sometimes seen close to fast destaining synapses. High resolution images of two such closely adjacent synapses are shown in Figure 2D. The delayed synapse (#2) showed almost no destaining after 5 s, when its faster neighbor (#1) had already released >50% of its initial intensity. The release kinetics were very similar when destaining finally set off, 6 s after stimulation start.

### Repetitive FM1-43 Loading and Unloading Modifies the Relative Proportion Between Kinetic Classes

**FIGURE 3:**
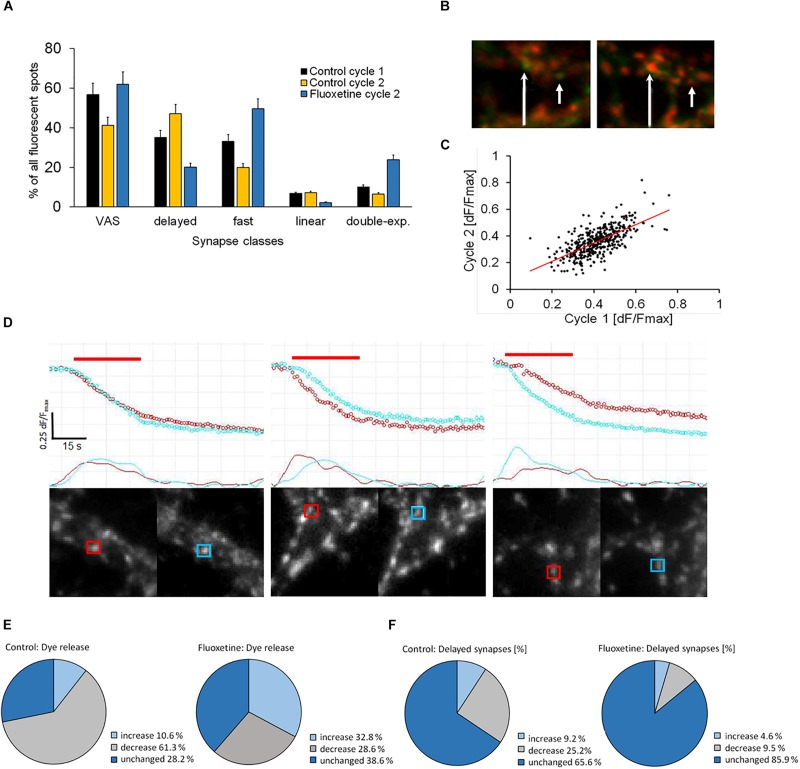
**(A)** Quantification of specified kinetic synapse classes in two subsequent staining/destaining cycles and effect of fluoxetine on class change in between both stimulation cycles (9 control experiments, 10,569 synapses; 11 fluoxetine experiments, and 11,299 synapses). ANOVA analysis indicated significant (*p* < 0.001) differences between the three groups in all five experimental setups. **(B)** Green color indicates staining of synapses with FM1-43, while red color shows direct subsequent stimulation-dependent destaining. Two single synapses (arrows) shows no destaining during the 1st stimulation cycle but recovered, and destained in the 2nd cycle. **(C)** Correlation plot of FM1-43 release between the 1st and the 2nd staining/destaining cycles (*n* = 424 matched synapses). **(D)** Direct comparison of individual synapses in subsequent staining/destaining cycles. Upper traces show the course of fluorescence and lower traces their corresponding first-order derivatives. First cycle (red), 2nd cycle (cyan), and stimulation period (red bar). The image pairs and the traces were directly obtained with SynCom Ver. 1.61, software. **(E,F)** The pie-charts display the differences between two successive staining/destaining cycles when no drug (control) or 10 μM fluoxetine was administered between the cycles, control *n* = 424, fluoxetine *n* = 387 synapses. **(E)** Differences in FM1-43 dye release from synapses between 1st and 2nd stimulation cycle. **(F)** Variances in the percentage of synapses with delayed release onset (DelSys) between 1st and 2nd stimulation cycle.

We investigated whether specific synaptic classes remained kinetically stable after repeated staining and destaining cycles, a procedure that subjected them to considerable stress. The experimental protocol was presented in Supplementary Figure S1 and described in detail previously (Henkel et al., 2010). During the second cycle, control preparations showed that the proportion of DelSyns was significantly increased, while the proportion of VAS was significantly reduced (*p* < 0.01) (Figure 3A). The same reduction was also observed in double – exponential – and fast synapses. In order to test whether these changes were related to pathological intra-synaptic mechanisms, fluoxetine was applied between the first and the second stimulation cycles. Fluoxetine, a classical SSRI-class anti-depression drug, was found to reduce excitotoxicity and to increase the mobilization of synaptic vesicles (Kim et al., 2013; Jung et al., 2014). Application of fluoxetine reversed and even over-compensated these kinetic changes. Figure 3A shows that fluoxetine enhanced the fraction of both fast releasing- and double exponential synapses, despite the repetitive staining–destaining cycle. The relative proportion of slow releasing synapses (DelSyns and “linear synapses”) was significantly reduced by >50% (*p* < 0.01). Figure 3B shows an example of two synapses that did not destain during the first stimulation cycle, but became active during the second cycle after the application of fluoxetine. These observations suggest that individual synapses may change their release kinetics in response to external stimuli or pharmacological interventions. When the release quantity was compared between both cycles, it was found that d*F*/*F*_*max*_ was a little (14.7%), but significantly (*p* < 0.01) reduced and poorly correlated (*r* = 0.66). The low correlation points to a high variability of release activity within an individual synapse, rather than toward an activation of previously silent synapses (Figure 3C).

Next, we checked whether this variability was mostly due to a change in the kinetic release mode of the same synapse. Therefore, individual synapses were localized and matched between the first and the second stimulation cycles and their release kinetics were directly compared. Figure 3D shows typical traces from three individual synapses that were identified and matched between two successive staining/destaining cycles (first cycle in red, second cycle in cyan). The two lower traces represented the filtered first-order derivatives of the upper ones. The left panel shows a fast responding synapse that largely retained its release kinetics during the second cycle. The middle panel shows a synapse that showed a delayed onset of release during the second cycle, while the synapse on the right panel showed a fast-responding one during the second cycle.

The proportion of dye release was very variable between two successive stimulation cycles. Less than a third of all synapses showed identical release proportions (d*F*/*F*_*max*_) in both cycles while almost two-thirds showed reduced exocytosis (Figure 3E). A few synapses (∼10%) released more dye in the second cycle, indicating a facilitation effect. Treatment with fluoxetine before the 2nd cycle caused synaptic facilitation in about a third of all synapses, while far fewer synapses reduced release compared to untreated synapses during the 2nd cycle. About 40% of the synapses performed the same in both cycles.

Figure 3F shows that the onset of release (*D*_*t*_) was delayed in some synapses to a variable degree. Although the majority (65%) of active individual synapses didn’t change their release kinetics between consecutive staining/destaining cycles, we found deviations when synapses were classified according to *D*_*t*_. In order to clearly separate groups, synapses were considered as “Changed,” if their individual *D*_*t*_ – change between the 1st and the 2nd cycle was longer than 2 s. Fluoxetine significantly increased the proportion of unchanged synapses when it was compared to controls (*Z*_*score*_ = −6.55, *p* = 0.01). Thus we tested if individual DelSyns retained their prolonged *D*_*ts*_ during the second stimulation cycle. When we compared active synapses with a delay of 3 or more seconds, we found that the vast majority (>90%) remained unchanged or decreased the delay times, despite the fact that the total number (Figure 3A) of DelSyns was increased in the second cycle. This indicated that previously fast responding synapses showed a delayed exocytosis onset. Fluoxetine retained the delay time between both cycles to the largest extent and reduced the proportion of delay-changing synapses distinctively. *D*_*ts*_, measured in DelSyns during the second stimulation cycle, were slightly reduced in response to fluoxetine. However, this reduction was not statistically significant (control: *D*_*t*_ = 4.48 ± 2.05, *n* = 172; fluoxetine: *D*_*t*_ = 4.1 ± 1.23, *n* = 56). The size effect was also small (Cohen’s *D* = 0.226).

### Delayed Onset of Exocytosis Is Not Caused by Delayed Calcium Entry

**FIGURE 4:**
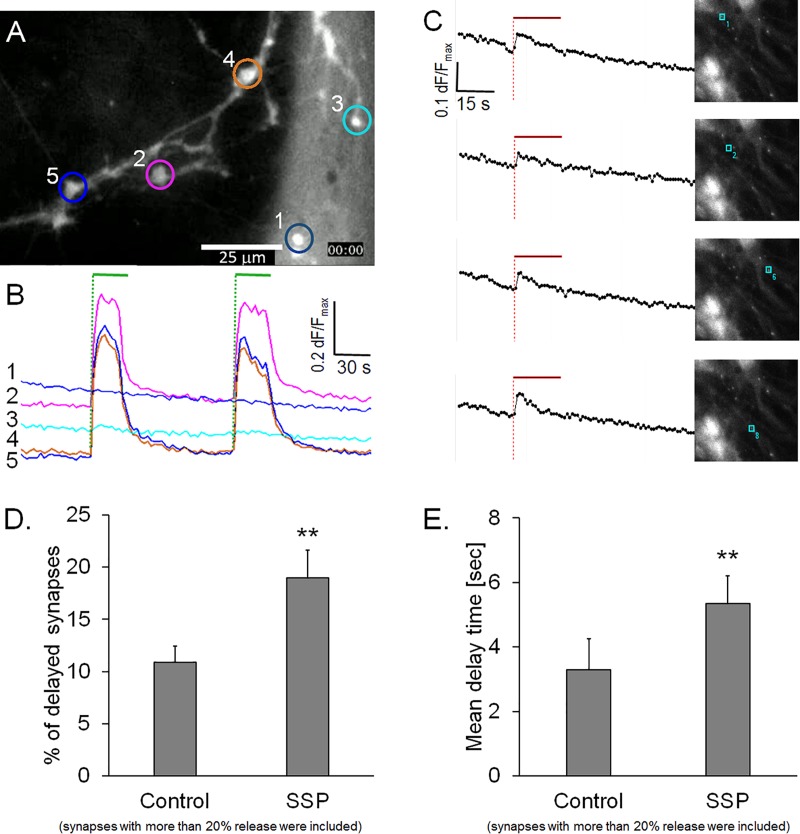
**(A)** Synapses loaded with calcium sensor Ca-green AM. **(B)** Traces from five regions in panel **A**, green bars: electrical field stimulation. **(C)** Synapses loaded with calcium sensor Fura-2 AM, traces are from single synapses in the corresponding images, red bar indicates electrical field stimulation. **(D)** Quantification of staurosporine (SSP)-treated preparations, percentage of active synapses (d*F*/*F*_*max*_ > 0.2), labeled with FM1-43 labeled, control: *n* = 3758, from five independent experiments, SSP: *n* = 4865, from six independent experiments. **(E)** Corresponding weighted mean delay times. Asterisks indicate a significant difference between SSP and control (*p* < 0.01).

Calcium has be shown to regulate the speed and magnitude of exocytosis (Neher and Sakaba, 2008); therefore, we investigated whether the delay in FM1-43 release was associated with a delayed calcium entry. We conducted experiments on hippocampal neurons that were preloaded with either Ca-Green AM or Fura-2 A, two well-known calcium sensors. Figure 4A shows a typical preparation that was stained with Ca-green AM and subjected to two successive stimulation cycles. None of the active synapses (*n* = 465) showed a delay after the start of the stimulation within our time resolution of 1 s (Figure 4B). Similarly, synapses loaded with Fura-2 AM showed an invariably sudden onset of synaptic calcium influx that started to decay even during the course of the stimulation (Figure 4C). These observations excluded the possibility that a delayed calcium entry could account for the observed delayed release of FM1-43 in a sub-population of synapses. It is noteworthy that our experimental setup did not allow the measurement of calcium in micro-domains inside synapses which could have accounted for high localized calcium concentrations.

### Staurosporine Increases the Proportion of Delayed Synapses in High-Releasing Synapses

We then addressed the question of whether a modulated opening kinetics of the fusion pore could cause the observed delay of FM1-43 release. There is evidence in several exocytosis model systems that the non-specific protein kinase inhibitor staurosporine inhibits fusion pore expansion and prevents vesicle collapse into the plasma membrane (Klingauf et al., 1998; Henkel et al., 2001; Rudling et al., 2018). FM1-43 staining of staurosporine-treated preparations was indistinguishable from controls (Cohen’s *D* = 0.14). Staurosporine led to a significant reduction of dye release from active synapses (16.1%, Cohen’s *D* = 4.8) and resulted in a significantly slower half time of release (tau_50_ = 10.0%) (Cohen’s *D* = 1.07). Furthermore, staurosporine treatment significantly increased the proportion of delayed synapses by 58.6%, when active synapses with release rates >0.2 d*F*/*F*_*max*_ were analyzed (Figure 4D). This result was even more pronounced, when all synapses (including low quantity releasing synapses) were included (data not shown). The weighted mean delay time (*D*_*t*_) was also 61.7% slower (Cohen’s *D* = 2.22) in staurosporine-treated synapses, compared to controls (Figure 4E). These observations support the hypothesis that inhibition of fusion pore expansion might be the underlying mechanism of delayed FM1-43 release.

### Synaptic Vesicle Exocytosis Is Not Delayed in SynaptopHlourin-Transfected Neurons

**FIGURE 5:**
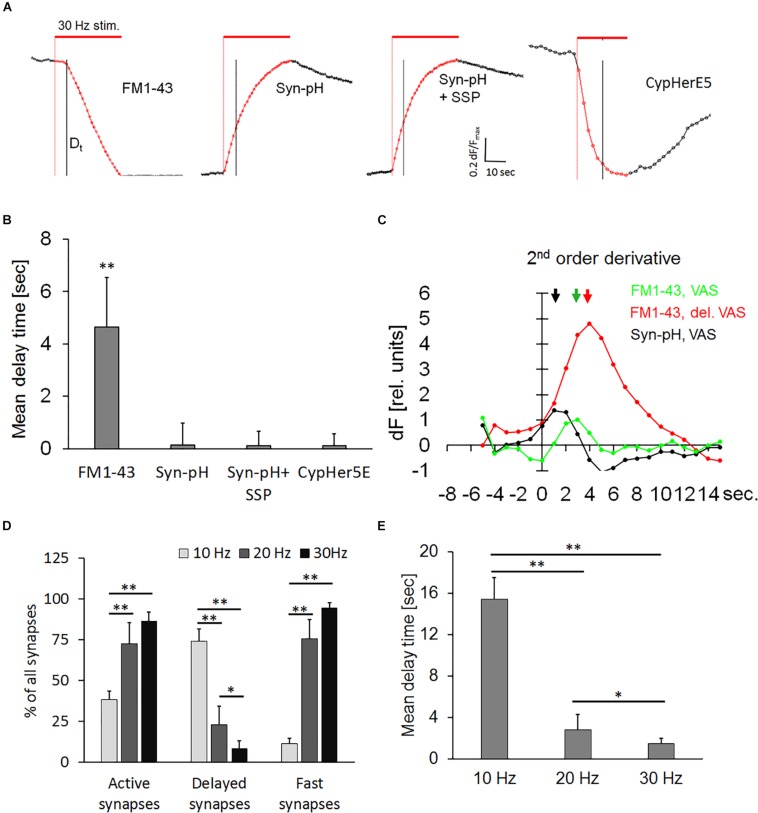
**(A)** Normalized averaged synaptic exocytosis traces from active synapses. Normalization was performed by stretching each individual trace between the start image and the first image after the end of stimulation to neutralize brightness variability and enhance the kinetic course. The red bar indicates electrical field stimulation at 30 Hz and the black vertical line the 5th second after the end of stimulation. Left panel: DelSyns, (FM1-43), subsequent panels as indicated: synaptopHlourin transfected synapses (Syn-pH), synaptopHlourin transfected synapses incubated with staurosporine (Syn-pH + SSP), and αSyt1-CypHerE5 labeled synapses (CypHer5E). **(B)** Quantification of weighted mean delay times [FM1-43: *n* = 1994 synapses (nine experiments), Syn-pH: *n* = 354 (six experiments), Syn-pH + SSP: *n* = 398 (five experiments)]. Syn-pH values were below the noise level in the automated SynoExa measurements, ANOVA analysis *p* < 0.001, the asterisk indicates a significant difference (*p* < 0.01) between FM1-43 and all other experimental conditions. **(C)** Second-order derivative traces (long-pass filtered) of VAS fluorescence, color-coded as indicated in the graph. The arrows point to the time positions of maximal speed change that correspond to the onset of exocytosis (*D*_*t*_). **(D)** Percentage of synapse classes at stimulation with three different frequencies: independent experiments at: 10 Hz *n* = 5, 20 Hz *n* = 5, 30 Hz *n* = 4; number of synapses at 10 Hz *n* = 2066, 20 Hz *n* = 2092, 30 Hz *n* = 1534 synapses, ANOVA analysis *p* < 0.001; asterisks indicate statistically significant difference (Student’s *t*-test, ^∗∗^*p* < 0.01, ^∗^*p* < 0.05). **(E)** Weighted mean delay times from active synapses (five independent experiments for each frequency) at indicated stimulation frequencies showed a significant increase of *D*_*t*_ at 10 Hz (*p* < 0.01) and at 20 Hz (*p* < 0.05) compared to 30 Hz.

The use of pH-sensitive dyes provides direct visual evidence on the existence of a hydrophilic connection (fusion pore) between the extracellular space and the vesicular lumen (Sankaranarayanan and Ryan, 2000). Thus, we transiently transfected hippocampal neurons with the pH-sensor synaptopHlourin, to test whether the delayed release phenomenon was due to a transient arrest of exocytosis or if a narrow fusion pore was formed, which could prevent the escape of FM1-43 from the vesicle. Transfected neurons and FM1-43-stained preparations were stimulated in seven independent experiments. All traces were normalized, since the synaptopHlourin signal increase was much weaker than the magnitude of FM1-43 destaining. Figure 5A shows normalized synaptic exocytosis traces and their corresponding quantification (Figure 5B). Active synapses stained with FM1-43 had a weighted mean *D*_*t*_ of 4.64 ± 1.9, seconds, *n* = 1994, while synaptopHlourin-transfected synapses showed no apparent delay (Figures 5A,B). Application of staurosporine did not change the instantaneous exocytosis onset in transfected synapses. Second-order derivatives of exocytosis traces, representing exocytotic velocity change during the stimulation period, showed that synaptopHlourin transfected VAS showed an immediate response to the stimulus and were distinctively faster than VAS stained with FM1-43 (Figure 5C). These observations suggest that an intravesicular pH equilibration with the extracellular fluid occurred instantaneously upon stimulation, most likely through a membrane spanning fusion pore.

### Recycled Vesicles, Labeled With Syt1-CypHerE5 Show No Delay

In order to test whether recycled vesicles were directed into a transient reserve pool that could potentially prolong a direct re-use, we permanently labeled them with a pH-sensitive antibody αSyt1-CypHerE5 (CypHer) against the inner vesicular domain of synaptotagmin. FM1-43 and CypHer shared the same labeling mechanism, namely the uptake of the probe during a complete cycle of exo/endocytosis but recycled vesicle could mix with other functional pools upon repetitive stimulation. This experiment could ascertain whether a transient fusion pore existed that allowed fast equilibration of lumenal vesicular pH. In clear contrast to observations on FM1-43-stained synapses, none of the active CypHer-labeled synapses showed a delay after the start of stimulation. This immediate response to stimulation was not affected by staurosporine, demonstrating that recycled vesicles were directly available for reuse (Figures 5A,B). The contrast between the dynamic of FM1-43 and CypHer is to their fundamentally different reporter mechanisms. Indeed, FM1-43 dye can depart from the vesicular membrane only after complete fusion, while the CypHer dye needs only a transient fusion pore to destain.

### Lower Frequency Stimulation Increases the Percentage and Delay Time in Synapses

The effect of stimulation frequency on delayed release of FM1-43 was determined to analyze whether the level of delay (kiss-and-run) and of full collapse are modulated by neuronal activity. Therefore, synapses were bleach-corrected and normalized before analysis. Figure 5D showed a significant increase in the percentage of delayed synapses and a corresponding decrease of fast-onset synapses at 10 Hz frequency compared to our standard stimulation protocol (30 Hz). Twenty and 30 Hz did not change the percentage of fast-onset synapses significantly. The percentage of active synapses was decreased to about 50% at 10 Hz, indicating that the release probability was slowed down compared to higher frequency stimulations. Figure 5E showed that the mean delay time was significantly (*p* < 0.01) longer at 10 Hz, compared to both 30 and 20 Hz, while *D*_*t*_ at 30 Hz was significantly (*p* < 0.05) shorter than at 20 Hz.

## Discussion

Synapses have been classified according to a variety of neurotransmitter types, release kinetics, and plasticity (Castillo, 2012; Emes and Grant, 2012; Nusser, 2018). However, it was still unclear whether all synapses of a defined type are functionally homogeneous or whether they exhibit individually distinct release kinetics. In the present work, we provide experimental evidence that synapses within a given neuron show heterogeneous behaviors. Thus, synapses can fine-tune their exo-endocytosis kinetics independent from their immediate neighboring synapses. Indeed, individual synaptic activity seemed to be governed by intra-synaptic factors. Since the experiments were conducted on neurons from newborn rats, it could not be excluded that neurons from adult animals would express a different activity.

We observed that prevalent synaptic exocytosis kinetics, like single exponential release functions, were the result of averaging of many synapses, while some individual synapses employed bi-phasic or linear release kinetics. It appears that complex synaptic networks might include a temporal component for information transfer and processing which involves dynamic change from fast to slow synapses and vice versa, adding a further dimension to the system of signal processing (Sakaba, 2008; Gu et al., 2012; Somogyi et al., 2014).

To our knowledge, this is the first report that documented a delayed onset of FM1-43 release of a few seconds in a sizable fraction of synapses. Therefore, we had to exclude experimental artifacts that may have been imposed by field stimulation inhomogeneity or imprecise timing. We did not find evidence that the local distribution of delayed synapses was correlated to the strength of the depolarizing field gradient. The increased prevalence of delayed synapses on cell clusters and their absence on elongated structures argued strongly against this possibility (Supplementary Figure S2). If a strength gradient would have been present, it would have shown an inhomogeneous synapse distribution depending on the distance to the stimulation electrodes. The presence of DelSyns clusters suggested rather that an unknown local factor was the cause for the delay. A timing artifact could also be excluded by the observation that occasionally two directly neighboring synapses showed different onset of FM1-43 release (Figure 2D).

First, we asked whether delayed FM1-43-stained synapses belonged to a neurotransmitter-specific type of synapse. We matched individual synapses in two consecutive stimulation series, measured their release kinetics, and formed two kinetic groups: (1) “Delayed onset,” *D*_*t*_ = 3 s and (2) “Fast onset,” *D*_*t*_ = 1 s. Even though this procedure underestimated the real number of changed synapses, the results suggested it was unlikely that a neurotransmitter-specific synapse was associated with a unique release mode, when about 35% could change their individual *D*_*t*_ by >2 s, either toward slower or faster release onset. It is noteworthy that the absolute percentage of synapses depended on the definition of “delayed onset.” Slightly different timing definitions may result in different absolute values. However, the original data were always compared to their respective controls by unbiased computer-based techniques, which provide stronger validation to our observation.

A lower stimulation frequency (10 Hz) increased the percentage delayed synapses while increasing their delay time, pointing to an activity regulated general mechanism of neurotransmitter release control. We did not observe a correspondingly large effect at 20 Hz compared to 30 Hz stimulation, an indication that stimulation frequency may not be directly correlated to the exocytosis mode and could be rather governed by non-linear calcium concentration (Blank et al., 2001). Taken together, these observations strongly suggest that at lower neuronal activity, synapses routinely work in a slow fusion pore expansion mode and accelerate toward the full fusion mode through a non-linear kinetic function (Wu et al., 2005).

Application of fluoxetine significantly stabilized the release mode, reduced the proportion of delayed synapses, and increased fast and bi-phasic synapses. This effect could be due to the increased size of the synaptic vesicle recycling pool and increased facilitation of exocytosis by fluoxetine (Jung et al., 2014). If our assumption was correct that delayed FM1-43 release onset was caused by the transient opening of a fusion pore that preceded the full fusion event, we could speculate that increased availability of vesicles for release shortened this pore transition toward full collapse. This, however, was not supported by our results. When *D*_*ts*_ of delayed synapses were compared between control and fluoxetine-treated preparation, we found no significant difference of individual *D*_*ts*_ or mean *D*_*ts*_ between both groups. Therefore, the effect of fluoxetine seems to be not directed onto the fusion pore kinetics itself, but rather to a reduced number of pores, capable of delaying full fusion. Previous studies have shown that fusion pore expansion was mediated by phosphorylation (Scepek et al., 1998; Fisher et al., 2001). Recent studies provided evidence that fluoxetine operates by enhancing the kinase activities of MZeta and Akt/GSK-3beta (Yan et al., 2017; Yi et al., 2018). It is plausible that fluoxetine effect on the release dynamics of synaptic vesicles seen in the present study is mediated through its kinase-enhancing activity. This could be investigated in detail in a set of future experiments where one would use more specific inhibitors for these kinases prior to fluoxetine application.

In the next sets of experiments, we tested whether the delayed FM1-43 release could be explained by either activation of different release modes or by shifts in vesicle pool dynamics. FM1-43 was the first widely successful optical probe to monitor the fusion of synaptic vesicles (Betz et al., 1992) in two complete consecutive cycles of exo-endocytosis (1st staining – 2nd destaining cycle). This mechanism labeled exclusively recycled vesicles but excluded vesicles from other pools and newly synthesized ones. Using FM1-43, stimulation-dependent exocytosis was only measurable, when the stained vesicle fused completely with the plasma membrane, but remained undetectable, if the vesicular lumen was only transiently connected to the extracellular space through a fusion pore (Richards et al., 2005). Although the mean FM1-43 release from a stimulated hippocampal neuronal preparation could be fitted with a single exponential decay function (Ratnayaka et al., 2012), we observed that this was due to an averaging effect of fast and slow destaining synapses. Some individual synapses showed a complete block of any destaining for up to several seconds after the start of electrical field stimulation. Calcium measurements, using two different sensor probes, showed consistently an instantaneous rise of Ca^2+^ in all imaged synapses without any delay, excluding the hypothesis that quiescent calcium channels were responsible for the effect. We then investigated two other possible hypotheses, namely the transient parking of newly recycled vesicles in a recycling pool that delayed their re-use and the formation of a transient fusion pore, since a full-collapse fusion was a prerequisite for FM1-43 release. To investigate the fusion pore hypothesis, cells were transfected with synaptopHlourin, a pH-sensitive reporter molecule that labeled pool-independent all vesicles. These experiments showed no delayed exocytosis onset in any of the monitored synapses, supporting the instantaneous formation of an aqueous channel between the vesicular lumen and the extracellular space. We then tested whether lingering in the recycling pool did transiently prevent the utilization of recycled vesicles for subsequent exocytosis. The recycling vesicle pool was specifically stained with Cypher, an antibody against the lumenal domain of the vesicle marker synaptotagmin. In contrast to FM1-43, this probe reported exocytosis by increased fluorescence upon pH increase that occurred when a narrow fusion pore on the plasma membrane was formed. Like synaptopHlourin, CypHer labeled synapses never showed a delayed onset of exocytosis, excluding a temporary hold of vesicle translocation to the fusion site. It also supported the hypothesis that a fusion pore was immediately formed after stimulation had started. This observation was in line with the results of experiments where single vesicles were labeled with quantum dots, suggesting a prolonged fusion pore connection that preceded full-fusion events of vesicles (Zhang et al., 2009). This observation was also made on hippocampal large dense core vesicles that released neuropeptides from sites on neuronal somata (Xia et al., 2009).

The next test for the temporary fusion pore hypothesis was the application of staurosporine that inhibited exocytosis by preventing the full collapse vesicle fusion (Henkel and Betz, 1995; Klingauf et al., 1998; Henkel et al., 2000, 2001). Although this mechanism has been challenged later in neuromuscular synapses (Becherer et al., 2001). In our experiments with FM1-43, staurosporine significantly increased the proportion of delayed synapses and extended the onset of dye release, supporting the idea that a transient fusion pore was the cause of the observed delay. This conclusion was supported by the observation that synaptopHlourin (proton-sensitive probe) never showed a delay either in the presence or the absence of staurosporine. In this model, the vesicles in delayed synapses immediately opened, as demonstrated in experiments with synaptopHlourin and Cypher. The pore expanded to full collapse fusion after few seconds, and released FM1-43.

Many studies have shown consistently that the staurosporine inhibited intrasynaptic vesicle movement (Kraszewski et al., 1996; Becherer et al., 2001; Jordan et al., 2005). Assuming that vesicle translocation toward the active zone was restricted by staurosporine, one would not expect a fast response to stimulation as it was always observed with synaptopHlourin and CypHer, independent of drug application. Movement-restriction by staurosporine would have prevented the translocation of CypHer-labeled recycled vesicles from a distant recycling pool to the active zone. Therefore, it appears likely that the majority of recycling took place very closely to the active zones and required a full collapse, followed by endocytosis. This mechanism is plausible because the uptake of large CypHer antibodies into vesicles through a small fusion pore was very unlikely because of their small diameter (Wu et al., 2016; Chang et al., 2017). Limitations with regard to relatively non-specific effects of staurosporine could be overcome by future analysis in synaptotagmin-7 knock-out neurons. Indeed, it has been shown that this protein may be responsible for repetitive open–close cycles of a fusion pore in chromaffin cells. This could prevent full fusion of synaptic vesicles (Rao et al., 2014).

**FIGURE 6:**
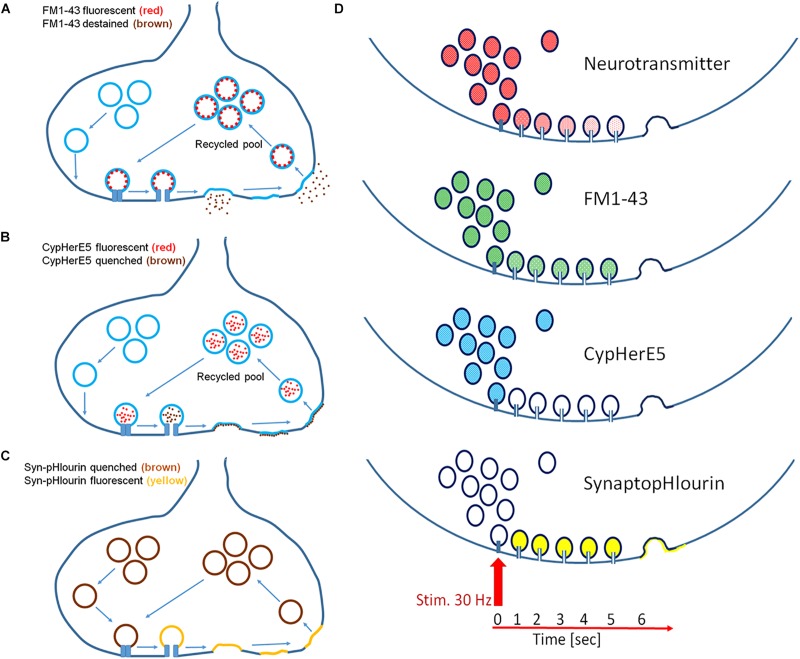
Mechanistic models of exocytosis monitoring. **(A)** FM1-43 labels exclusively recycled vesicles and its fluorescence destains only after full fusion but not upon fusion pore opening. **(B)** CypHerE5 permanently labels exclusively recycled vesicles and its fluorescence is transiently quenched upon fusion pore opening and restored after endocytosis and re-acidification of the vesicle. Recycled vesicle may become part of other functional pools upon repetitive stimulation. **(C)** SynaptopHlourin (Syn-pHluorin) labels all vesicles and reports fusion pore openings by increased fluorescence. Endocytosis quenches fluorescence reversible after re-acidification of the vesicle. **(D)** Proposed models of exocytosis from an individual vesicle: neurotransmitter is slowly released through a narrow fusion pore under the premises that it is partially sequestered onto a intravesicular matrix, FM1-43 is not released through a narrow pore, CypHerE5 (darkening) and SynaptopHlourin (brightening) indicate instant opening of the pore.

In this study we used several different fluorescent probes to propose a mechanistic model that was in line with our observations (Figures 6A–D). While FM1-43 and CypHer antibody did exclusively stain recycled vesicles, synaptopHlourin labeled the whole pool (Betz and Bewick, 1992; Sankaranarayanan and Ryan, 2000; Adie et al., 2002). CypHer and synaptopHlourin reported “*classical exocytosis*” and “*kiss-and-run*” by visualizing a breakdown of an intravesicular pH gradient, while FM1-43 indicated only full collapse fusion by its dissociation from the vesicle membrane. Since CypHer became permanently attached to the inner vesicular membrane after initial contact, it acted as a permanent reporter for repetitive vesicle-fusion pore-plasma membrane interactions. Finally, inner-synaptic transport pathways between a spatially separated recycling pool and release sites could be monitored by FM1-43 and CypHer.

Delayed release of FM1-43 in combination with immediate fluorescence loss of CypHer and instantaneous increase of synaptopHlourin fluorescence point to the instantaneous calcium-triggered formation of a transient fusion pore, followed by full exocytosis and subsequent endocytosis. The staurosporine data support this interpretation if one assumes a general vesicle mobility inhibition by this drug required a local recycling mechanism for CypHer. The other proposed action of staurosporine, namely the promotion of “*kiss-and-run*” release, explains the appearance of more delayed synapses and extended delay times (*D*_*t*_). Taken together, our data support the idea that an instantaneous opening of a fusion pore allowed neurotransmitter release and proceeded later toward “full collapse vesicle fusion,” followed by endocytosis. The time interval between the first opening and the subsequent full fusion can be regulated by neuronal activity and kinase-modulating chemicals. Our suggestion that the differential opening characteristics of a fusion pore was the main cause for the observed effects is only indirect, since we could not study individual vesicle fusion. However, very recently it was observed that individual synaptic vesicles undergo several kiss-and-run events within 10 s after the start of stimulation and finally fuse completely with the plasma membrane (Qin et al., 2019). These recent findings are in complete agreement with our mechanistic model and provide further support to our conclusions. In summary, in addition to the classical quantitative regulation of synaptic transmission, we provide experimental evidence to support a novel process by which inter-synaptic information flow can be temporally modulated.

## Data Availability Statement

The datasets generated for this study are available on request to the corresponding author.

## Ethics Statement

The animal studies were reviewed and approved by the Kollegiales Leitungsgremium of the Franz-Penzoldt Zentrum, Erlangen, Germany, in accordance with the Animal Protection Law of the Federal Republic of Germany and by the Kuwait University Research Administration Ethics Committee.

## Author Contributions

AH designed and performed the experiments. AH and AM coordinated the experiments, analyzed the data, and co-wrote the manuscript. OW designed and conducted the experiments.

## Conflict of Interest

The authors declare that the research was conducted in the absence of any commercial or financial relationships that could be construed as a potential conflict of interest.

## Supplementary Material

FIGURE S1 Sequential stain exocytosis assay for hippocampal neurons, modified from Henkel et al. (2010). Hippocampal neurons were stained with FM1-43 in the absence of fluoxetine, washed, and pictures were taken during the period, indicated by dots. Fluoxetine was added (red line) after the first staining/destaining series and remained in the bath during the further rest of the experiment. The staining/destaining procedure was repeated in a second series in presence of the drug and pictures were taken. These two resulting image series were subsequently compared and analyzed.

FIGURE S2 **(Top)** FM1-43-stained hippocampal synapses. **(Bottom)** Distribution of active synapses (green), delayed active synapses (red).
