# Supplementary figures and images for: Differential Release of Exocytosis Marker Dyes Indicates Stimulation-Dependent Regulation of Synaptic Activity
Source: Front Neurosci. 2019 Oct 2;13:1047. doi: 10.3389/fnins.2019.01047 (PMC6783566; doi:10.3389/fnins.2019.01047)

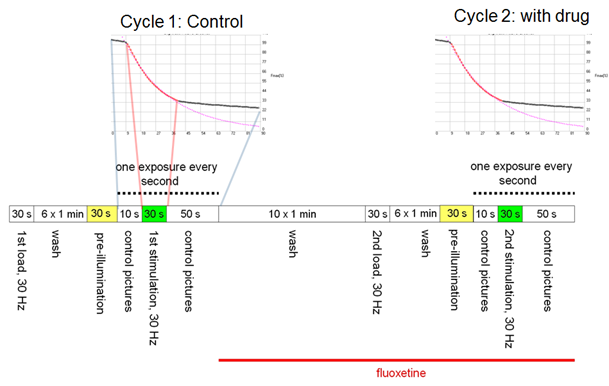

Supplement: FIGURE S1 — Sequential stain exocytosis assay for hippocampal neurons, modified from Henkel et al. (2010). Hippocampal neurons were stained with FM1-43 in the absence of fluoxetine, washed, and pictures were taken during the period, indicated by dots. Fluoxetine was added (red line) after the first staining/destaining series and remained in the bath during the further rest of the experiment. The staining/destaining procedure was repeated in a second series in presence of the drug and pictures were taken. These two resulting image series were subsequently compared and analyzed. [file Image_1.TIF]

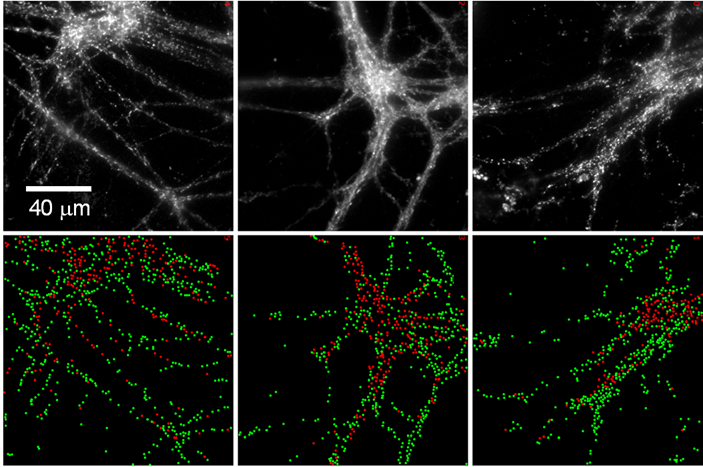

Supplement: FIGURE S2 — (Top) FM1-43-stained hippocampal synapses. (Bottom) Distribution of active synapses (green), delayed active synapses (red). [file Image_2.TIF]
